# NR4A3: A Key Nuclear Receptor in Vascular Biology, Cardiovascular Remodeling, and Beyond

**DOI:** 10.3390/ijms222111371

**Published:** 2021-10-21

**Authors:** José Martínez-González, Laia Cañes, Judith Alonso, Carme Ballester-Servera, Antonio Rodríguez-Sinovas, Irene Corrales, Cristina Rodríguez

**Affiliations:** 1 Instituto de Investigaciones Biomédicas de Barcelona-Consejo Superior de Investigaciones Científicas (IIBB-CSIC), 08036 Barcelona, Spain; lcaees@gmail.com (L.C.); jalonson@santpau.cat (J.A.); cballester@santpau.cat (C.B.-S.); 2 CIBER de Enfermedades Cardiovasculares, ISCIII, 28029 Madrid, Spain; antonio.rodriguez.sinovas@vhir.org; 3 Instituto de Investigación Biomédica Sant Pau, 08041 Barcelona, Spain; 4 Cardiovascular Diseases Research Group, Vall d’Hebron Institut de Recerca, Vall d’Hebron Hospital Universitari, Vall d’Hebron Barcelona Hospital Campus, 08035 Barcelona, Spain; 5 Laboratorio de Coagulopatías Congénitas, Banc de Sang i Teixits (BST), 08005 Barcelona, Spain; icorrales@bst.cat; 6 Medicina Transfusional, Vall d’Hebron Institut de Recerca-Universitat Autònoma de Barcelona (VHIR-UAB), 08035 Barcelona, Spain; 7 Institut de Recerca Hospital de la Santa Creu i Sant Pau (IRHSCSP), 08041 Barcelona, Spain

**Keywords:** NOR-1, cardiovascular remodeling, atherosclerosis, abdominal aortic aneurysm

## Abstract

The mechanisms committed in the activation and response of vascular and inflammatory immune cells play a major role in tissue remodeling in cardiovascular diseases (CVDs) such as atherosclerosis, pulmonary arterial hypertension, and abdominal aortic aneurysm. Cardiovascular remodeling entails interrelated cellular processes (proliferation, survival/apoptosis, inflammation, extracellular matrix (ECM) synthesis/degradation, redox homeostasis, etc.) coordinately regulated by a reduced number of transcription factors. Nuclear receptors of the subfamily 4 group A (NR4A) have recently emerged as key master genes in multiple cellular processes and vital functions of different organs, and have been involved in a variety of high-incidence human pathologies including atherosclerosis and other CVDs. This paper reviews the major findings involving NR4A3 (Neuron-derived Orphan Receptor 1, NOR-1) in the cardiovascular remodeling operating in these diseases.

## 1. Introduction

Cardiovascular diseases (CVDs) are the leading cause of death globally, taking an estimated of more than 17 million lives each year (18.6 million (33.6% of all deaths worldwide) according to the 2019 Global Burden of Disease (GBD) Study update) [1]. Atherosclerosis, as the common pathological substrate of ischemic heart disease (IHD) and ischemic cerebrovascular disease, is responsible for a striking reduction in quality of life and life expectancy and imposes huge costs on health systems worldwide. The aging of the population has contributed to the increase of other chronic CVDs in which extensive tissue remodeling takes place, such as abdominal aortic aneurysm (AAA) and heart failure (HF). Cardiovascular remodeling is a complex response that involves several cell types and interrelated processes (inflammation, extracellular matrix (ECM) remodeling, altered calcium and redox homeostasis, apoptosis, etc.), activated by intricate gene programs orchestrated by a reduced number of transcription factors. Nuclear receptors (NRs) are master regulators of a plethora of cellular and biological processes such as cell proliferation, migration, apoptosis, metabolism, and differentiation. Recently, NRs of the NR4A subfamily have emerged as key players in the pathophysiology of CVDs. This paper reviews the major findings involving NR4A3 (NOR-1) in atherosclerosis and other CVDs.

## 2. Tissue Remodeling in CVDs

Atherosclerosis is a chronic inflammatory disease affecting medium and large arteries favored by risk factors such as hyperlipidaemia, hypertension, diabetes, or smoking. This disease is characterized by the progressive accumulation of lipids, inflammatory cells (macrophages, lymphocytes, dendritic cells, etc.) vascular smooth muscle cells (VSMC), ECM proteins, and calcium in the intimal layer [2]. Atherosclerosis arises as a result of the disturbance of endothelial homeostasis that allows the entry of plasma low density lipoproteins (LDL) into the subendothelial space. There, LDL are entrapped and chemically modified, mainly by oxidation, acquiring immunogenic properties [3]. This triggers a complex pathogenic process characterized by the increased expression of adhesion molecules, which promotes the recruitment of inflammatory cells such as monocytes. Recruited monocytes differentiate into macrophages and internalize oxidized LDL (oxLDL) becoming foam cells, which produce chemokines, cytokines, growth factors, and reactive oxygen species (ROS). At the same time, medial VSMC migrate into the intima, proliferate and produce ECM components [2]. This allows the grow of the atherosclerotic lesion and the formation of a fibrous cap covering the lipid-rich necrotic core composed of foam cells, cholesterol and apoptotic or necrotic cells. Exaggerated hyperplasia of pulmonary VSMC is also the main characteristic of vascular remodeling in pulmonary arterial hypertension, a severe and lethal condition that can lead to right ventricular failure [4]. By contrast, the loss of VSMC by apoptosis and degradation of ECM components are the main features of vascular remodeling in AAA, a life-threatening degenerative disease characterized by a progressive weakening and dilation of the arterial wall. Chronic inflammation, oxidative stress, neovascularization, apoptosis, and vascular calcification are some features common to both atherosclerosis and AAA [5,6]. Finally, adverse cardiac remodeling accompanies the development of HF and determines the clinical course of this disease characterized by the impairment of the ventricle’s blood filling and ejection capacity resulting from cardiac overload (due to aortic stenosis, hypertension, or valvular diseases) or injury (such as myocardial infarction) [7,8]. Ventricular remodeling encompasses molecular, cellular, and interstitial alterations that trigger changes in size, mass, geometry, and function of the heart leading to a decline in ventricular function and arrhythmias. The mechanisms underlying cardiac remodeling include cardiomyocyte cell death, altered energy metabolism, changes in contractile proteins, oxidative stress, inflammation, disturbed calcium handling, and fibrosis, among others. 

## 3. The NR4A Subfamily of NRs

The NR4A subfamily comprises three members: nerve growth factor-induced gene B (NGFI-B/Nur77 or NR4A1), nuclear receptor related protein 1 (Nurr1 or NR4A2), and neuron-derived orphan receptor 1 (NOR-1 or NR4A3, also known as TEC, MINOR or CHN). All three NR4A receptors exhibit the common structure of canonical NRs characterized by the presence of the N-terminal activation function-1 (AF-1) transactivation domain, a highly conserved DNA-binding domain (DBD) and a ligand-binding domain (LBD) located in their C-terminal region. A high degree of sequence homology among NR4A receptors is found in their DBD (90–95%), while N-terminal domains are fairly divergent [9]. Although they share the typical structure of NRs, NR4A receptors feature some specificities, the most relevant affecting the LBD. Canonical NRs bind small lipophilic molecules (ligands) thereby becoming active transcription factors. However, NR4A receptors seem to be constitutively-active orphan receptors, whose transcriptional activity is independent of the binding of a ligand. Indeed, NR4A receptors possess an atypical LBD structure, as supported by X-ray crystallographic analysis revealing the presence of tightly packed and large hydrophobic residues occupying the space that should correspond to the ligand-binding pocket [10,11]. Nonetheless, subsequent studies suggested the ability of the fungal compound cytosporone B to bind with high affinity the LBD of NR4A1 inducing the transcriptional activity of target genes [12]. Thereafter, several endogenous ligands have been proposed to directly bind and activate Nurr1-dependent transcription [13]. However, NR4A receptors remain considered constitutively-active NRs whose transcriptional activity depends on their gene expression levels, post-translational regulation, and interaction with co-regulatory partners.

**Figure 1:**
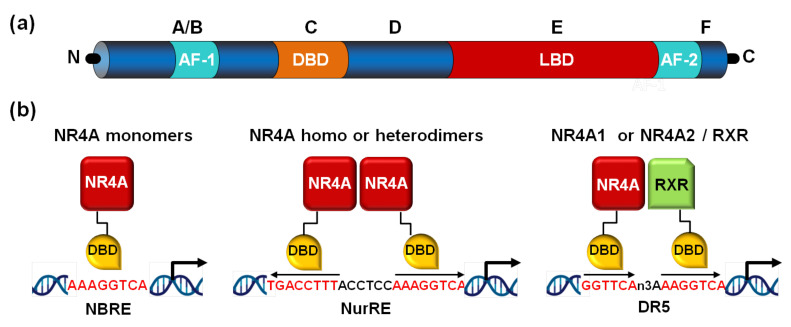
NR4A structure and binding to promoter response elements. (**a**) Nuclear receptors share a modular structure consisting of a variable N-terminal region, a DNA-binding domain (DBD) or region C, located in the central region, a variable linker domain (region D), and the E/F region in the C-terminal domain, which contains the ligand-binding domain (LBD). The ligand-independent activation function-1 (AF-1) and the ligand-dependent transactivation domain (AF-2) located in the N- and C-terminal regions, respectively, are also indicated. (**b**) NR4A receptors bind to specific DNA motifs in the promoter of their target genes. They bind as monomers to the NGFI-B response element (NBRE), as homodimers or heterodimers to the Nur-response element (NurRE), and NR4A1 and NR4A2 as heterodimers with retinoid X receptors (RXRs) to a DR5 motif.

NR4A receptors bind as monomers to a NBRE (Nerve growth Factor [NGFI-B] Response Element) consisting of an octameric sequence (consensus sequence AAAGGTCA). Further, as homodimers or heterodimers, they recognize a Nur-Response Element (NuRE) composed of two inverted NBREs spaced by a limited number of nucleotides [9]. However, NOR-1 shows low affinity for this response element, resulting in a reduced ability to activate gene transcription [14]. Likewise, Nur77 and Nurr1, but not NOR-1, can heterodimerize with retinoid X receptor (RXR) (Figure 1). These receptors can also indirectly modulate gene expression by crosstalk with other transcription factors. Indeed, they antagonize nuclear factor kappa B (NFκB) through diverse mechanisms including: binding with low affinity to NFκB sites [15], up-regulating the expression of the NFκB inhibitor IκBα [16], by physical association with p65 [17], or attenuating signaling pathways involved in NFκB activation [18]. Depending on the biological context, all three NR4A receptors can act cooperatively or exert antagonistic effects.

NR4A receptors are immediate-early genes, whose expression is rapidly induced in response to a plethora of stimuli [9]. Further, post-translational modifications, including protein phosphorylation [19], acetylation [20], or sumoylation [21] alter NR4A activity. These NRs are widely expressed. Tissues with high metabolic activity such as the brain, heart, skeletal muscle, adipose tissue, kidney, and liver display the highest expression levels of NR4A receptors [22,23]. Moreover, they are quickly induced by physiologic stimuli in cells and tissues in which their basal expression is low. Growth factors [22,24], native and oxLDL [18,25,26], angiogenic and inflammatory cytokines [18,27,28], hormones [29], thrombin [30,31], hypoxia [32,33,34], physical and mechanical factors [35,36], and neuronal membrane depolarization [37], among others, have been reported to enhance their expression or transcriptional activity. 

NR4A receptors closely exemplify the wide range of functions fulfilled by NRs, and have emerged as critical candidates for the coordinated regulation of multiple cellular processes (proliferation, differentiation, apoptosis, survival, inflammation, embryonic and neuronal development), lipid and carbohydrate metabolism [38,39,40], and vital functions for different organs, from the physiological adaptation to physical exercise to hepatic regeneration or memory [41,42,43,44]. This explains the involvement of NR4A receptors in a variety of high-incidence human pathologies such as CVDs [9,45,46], obesity [47,48], diabetes [49], cancer [50,51], and Parkinson’s disease [52] (Table 1).

### 3.1. NOR-1 in Atherosclerosis and Intimal Hyperplasia 

**Figure 2:**
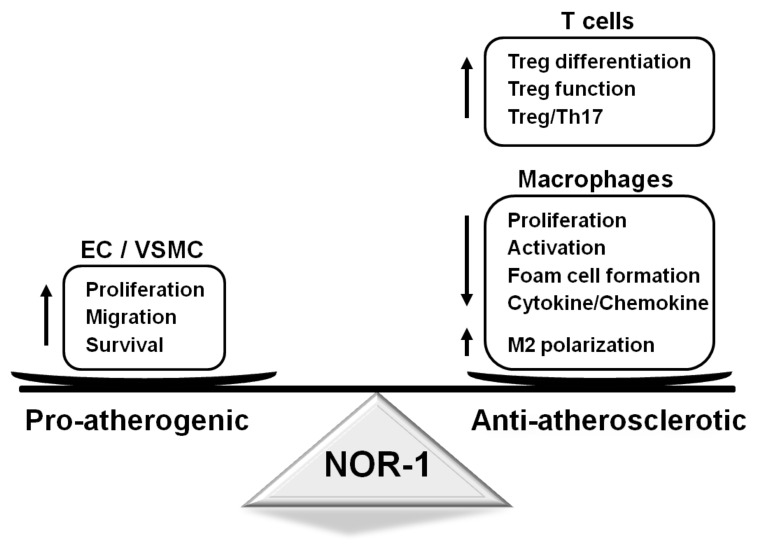
Functions regulated by NOR-1 in vascular and inflammatory cells and their impact on atherosclerosis. NOR-1 promotes vascular cell proliferation, migration, and survival, which might favor atherosclerosis progression. In turn, in T cells, this transcription factor controls Treg function and differentiation, while, in macrophages, NOR-1 induces M2 polarization, limits their activation, proliferation, and cytokine secretion and prevents foam cell formation. EC: endothelial cells; NOR-1: neuron-derived orphan receptor1; Th17: T helper 17 cells; Treg: regulatory T cells; VSMC: vascular smooth muscle cells.

Our studies at the beginning of this century revealed the up-regulation of NOR-1 in atherosclerotic plaques from patients with IHD and vascular lesions from pigs fed an atherogenic diet or subjected to percutaneous transluminal coronary angioplasty [22,53]. NOR-1, as well as Nur77 and Nurr1, is strongly induced by atherogenic stimulus in different cell types involved in atherosclerosis: VSMC, endothelial cells, monocytes-macrophages, and lymphocytes. Nevertheless, the first experimental approaches addressing the role of NOR-1 in vascular remodeling raised some controversy. These studies found that NR4A receptors are up-regulated in the neointima of human atherosclerotic plaques ranging from type II to V. Further insights in mice over-expressing a dominant-negative variant that suppresses the transcriptional activity of all three NR4A receptors led to the assumption that they all inhibit neointimal thickening [54]. Subsequent studies, however, ruled out a redundant role of these receptors in VSMC hyperplasia. Indeed, NOR-1 regulates key genes involved in cell cycle progression and its over-expression increases cell proliferation after vascular injury [22,24,25,28]. In contrast, Nur77 negatively modulates vascular cell proliferation [54,55,56]. This role of NOR-1 in intimal hyperplasia is clearly distinguished from its anti-inflammatory effect in chronic atherosclerosis (Figure 2). The experimental findings that have enabled a better understanding of the relationship between NOR-1 and the cellular and molecular mechanisms of vascular remodeling are detailed below. Additional information about NR4A receptors in the vascular wall can be found in previous review articles published by experts in the field [38,107,108,109].

#### 3.1.1. NOR-1 in Vascular Endothelial Cells

NOR-1 is expressed in endothelial cells and it is induced by stimuli which disturb endothelial function and foster endothelial cell migration, proliferation, and survival. High-throughput screening for differential gene expression identified NOR-1, and the other two members of the NR4A subfamily, among the most VEGF-induced genes in endothelial cells [64]. The expression of NOR-1 is strongly and transiently up-regulated in endothelial cells exposed to increasing concentrations of VEGF [28] or thrombin [30,31]. The effect depends on the activation of their respective receptors, the VEGF receptor-2 (VEGFR-2) and PAR-1, and involves several signaling pathways including increased cytosolic Ca^2+^ and activation of protein kinase C (PKC), as well as activation of extracellular signal-regulated kinase 1/2 (ERK1/2) and p38 mitogen-activated protein kinase (MAPK) pathways [28,30,31]. Notably, the blockade of NOR-1 using antisense oligonucleotides prevents both the migratory and the proliferative responses triggered by these stimuli. Interestingly, whole genome DNA methylation and transcriptomic analysis also identified NOR-1 as a gene induced during the angiogenic transformation of human brain microendothelial cells (HBMEC). The crucial role of NOR-1 in this process was evidenced by gene silencing with a CRISPR/Cas9 strategy, which limited cell migration and neovessell formation [66]. More recently, it has been shown that NOR-1 transcriptionally regulates endothelin-1 (ET-1) and mediates the pro-angiogenic response elicited by this vasoconstrictor peptide [67]. 

NOR-1 is further induced in endothelial cells exposed to hypoxia, primarily through a hypoxia-inducible factor 1 (HIF-1)-dependent mechanism involving an increase in both, cytosolic Ca^2+^ and the phosphatidylinositol 3-kinase (PI3K)/Akt/mTOR pathway [32], and the binding of HIF-1 to a Hypoxia Response Element (HRE) present in its proximal promoter region. This up-regulation of NOR-1 mediates the adaptive survival response of endothelial cells to hypoxia. NOR-1 inhibition leads to an increase in the percentage of apoptotic cells, while its over-expression promotes cell survival limiting apoptosis [32,33]. This pro-survival effect is triggered, at least in a part, by the NOR-1-dependent induction of cIAP2 (cellular inhibitor of apoptosis 2) [32,34], an anti-apoptotic protein whose transcriptional activity is regulated by NOR-1 through a NBRE [34]. Recent studies have revealed that NOR-1 activity is also modulated by post-translational mechanisms [110]. NOR-1 protein levels were dramatically decreased in endothelial cells subjected to reoxygenation after oxygen–glucose deprivation. The effect was blocked by the antioxidant TRIOL, which ameliorated endothelial hyperpermeability provoked by acute hypobaric hypoxia in vivo. The mechanism involves ROS-driven ubiquitination and degradation of NOR-1 promoted by SMARCB1 (SWI/SNF related, matrix associated, actin dependent regulator of chromatin, subfamily B, member 1) [110]. The authors highlighted the potential usefulness of these findings to design strategies that improve pulmonary endothelial barrier in acute lung injury.

Inflammatory stimuli (IL-1β, LDLox, lipopolysaccharide [LPS] and TNFα) also enhance the expression of NOR-1 in endothelial cells. This is associated with an induction of vascular cell adhesion molecule 1 (VCAM-1), whose expression is regulated by NOR-1 [57]. miR-17 and miR-20a would contribute to the NOR-1-dependent modulation of VCAM-1. These microRNAs (miRNAs) are expressed in the vascular wall and control NOR-1 mRNA levels in endothelial cells, thereby attenuating the expression of VCAM-1 [70]. Interestingly, NOR-1 deficiency, and the consequent reduction of endothelial VCAM-1, decreases the content of macrophages in atherosclerotic lesions in ApoE^−/−^ mice fed an atherogenic diet [57]. Further, both NOR-1 and the receptor for advanced glycation end products (RAGE) would also be involved in the pro-inflammatory and pro-atherogenic properties of glycated apolipoprotein A-IV (g-ApoA-IV), contributing to the vascular complications in diabetic patients [76]. In fact, g-apoA-IV, that induces inflammatory reactions in endothelial cells and atherosclerosis in ApoE^−/−^ mice, up-regulates endothelial NOR-1 expression. In turn, NOR-1 knockdown suppresses g-apoA-IV-dependent inflammation and atherosclerosis [76]. The expression of NOR-1 in the endothelium and microvessels of atherosclerotic plaques from diabetic patients supports the physiological relevance of these experimental findings. Finally, gain- and loss-of-function approaches indicates that NOR-1 modulates the expression of thrombomodulin [65], which is highly expressed on the surface of endothelial cells and exerts potent anti-coagulant, anti-fibrinolytic, and anti-inflammatory actions.

#### 3.1.2. NOR-1 in VSMC 

In quiescent VSMC, the expression of NOR-1 is extremely low, but it is strongly and quickly induced by multiple stimuli, including growth factors (platelet-derived growth factor [PDGF] and epidermal growth factor [EGF]), thrombin, native and oxLDL, and cytokines. Furthermore, this NR regulates the molecular mechanisms that control the migratory and proliferative activity of VMSC and their inflammatory response [18,22,24,25,53]. As described above, the induction of NOR-1 by these effectors relies on the activation of several signaling pathways that depend on the increase in cytosolic Ca^2+,^ and the activation of PKC and mitogen-activated protein kinases (ERK1/2 y p38 MAPK), among others. In turn, NOR-1 inhibition reduces the migratory and proliferative capacity of VSMC in culture [22,24,25]. The activation of all of these signaling pathways is required to achieve the maximal NOR-1 induction and the consequent high proliferative activity. Thus, the individual blockade of intracellular Ca^2+^ mobilization prevents NOR-1 induction and VSMC proliferation. A similar effect is produced when the intermediate conductance calcium-activated potassium channel KCa3.1 is blocked [59]. This approach inhibits NOR-1 expression and abolishes VSMC proliferation, allowing cells to keep their round shape associated with high l-caldesmon expression and low levels of calponin-1 (indicative of a de-differentiation state). Conversely, the activation of KCa3.1 induces NOR-1 and promotes the proliferation of VSMC, which acquire a spindle-shaped cellular aspect with low l-caldesmon and high calponin-1 [59]. Studies analyzing the impact of the cell shape per se on VSMC proliferation corroborate the critical reliance of NOR-1 on this process [111]. Restricting VSMC spreading in a single direction evokes cell elongation and reduces both NOR-1 expression and cell proliferation. NOR-1 knockdown studies further supported the fundamental role of NOR-1 governing vascular cell-shape and proliferation [111]. 

**Figure 3:**
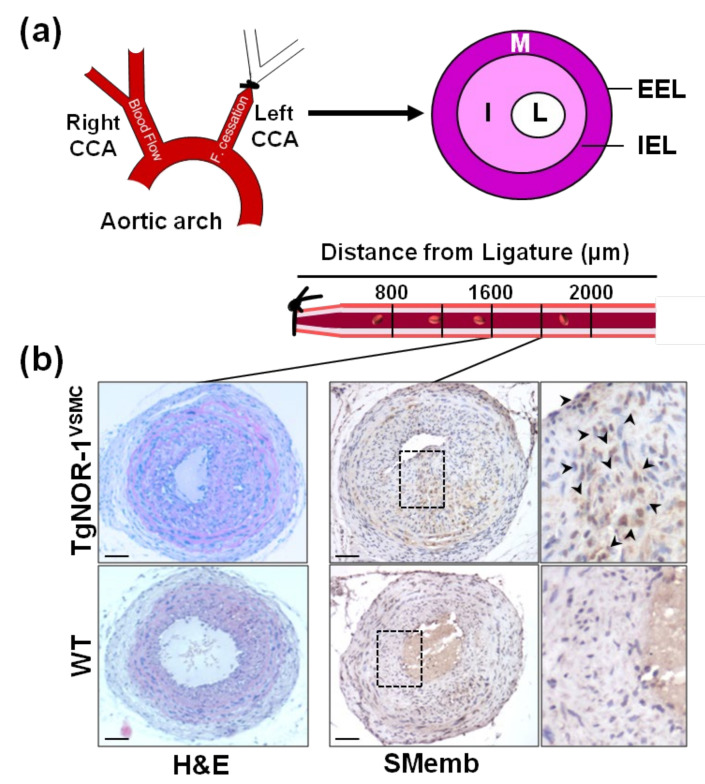
NOR-1 exacerbates neointimal thickening after vascular injury. (**a**) The scheme depicts neointimal hyperplasia induced by permanent ligation of the left common carotid artery (CAA). The specific regions analyzed based on their distance to the ligation site are indicated below (**b**) Hematoxylin-and-eosin (H&E) staining in sections at 1600 µm from the ligature evidences the stronger neointimal growth induced by carotid artery ligation in TgNOR-1^VSMC^ mice compared with WT littermates. NOR-1 transgenesis also exacerbates the vascular expression of SMemb, a marker of VSMC phenotype, strongly up-regulated in proliferating VSMC. Indicated areas are magnified on the right. EEL: External elastic lamina; IEL: Internal elastic lamina; I: Intima; L: Lumen; M: Media. Bars: 100 µm. This figure is based on the studies that led to previously published data [73].

The generation of genetically modified animal models has been crucial to improve our understanding about the role of NOR-1 in vascular biology. In this context, our group generated a transgenic mouse model over-expressing the human NOR-1 cDNA under the control of the transgelin (SM22α) promoter, which drives transgene expression specifically to SMC. We found that NOR-1 over-expression was associated with an exacerbated remodeling in response to carotid artery ligation, characterized by increased neointimal thickening [73,112]. Moreover, VSMC from transgenic mice exhibit higher proliferative rates and enhanced expression of embryonic smooth muscle myosin heavy chain/ myosin heavy chain 10 (Myh10/SMemb), a synthetic SMC marker up-regulated in proliferating VSMC (Figure 3) [113]. Subsequently, other authors confirmed our findings. Certainly, using the guidewire-induced arterial injury model in NOR-1 deficient mice, Nomiyama and collaborators found that NOR-1 is an essential transcription factor for VSMC proliferation, and identified cyclin D as a NOR-1 target gene [72]. Similarly, Dr. Bruemmer’s group determined that S phase kinase-associated protein 2 (SKP2), is also a transcriptional target for NOR-1 in VSMC [58]. Electrophoretic mobility shift and chromatin immunoprecipitation assays provided evidence that NOR-1 transactivates SKP2 promoter by binding to a NBRE site. SPK2, which is induced by growth factors, constitutes a limiting component of the ubiquitin ligase SCF complex (Skp1/Cul1/Fbox) and is responsible for the targeted recognition and degradation of several cyclin-dependent kinase inhibitors, including p27 [114,115]. It should be highlighted that post-translational phosphorylation increases NOR-1 activity in VSMC. Indeed, in human aortic VSMC, this NR forms a complex with the DNA-dependent protein kinase (DNA-PK) that phosphorylates NOR-1 in its N-terminal domain preventing its ubiquination and degradation [19]. Both NOR-1 and active DNA-PK co-localize in the neointima of carotid endarterectomy specimens, while the inhibition of DNA-PK limits neointimal thickening in a mouse model of arterial injury [19].

Altogether, these findings demonstrate that NOR-1 controls the expression of key elements involved in cell cycle progression, which explains the great impact of experimental interventions affecting NOR-1 expression on cell proliferation, vascular hyperplasia and remodeling. Consequently, anti-proliferative drugs dramatically reduce NOR-1 expression. The inhibition of VSMC proliferation by the anti-atherogenic drug simvastatin decreases NOR-1 expression both in vitro and in vivo. Certainly, simvastatin limited the LDL-mediated induction of NOR-1 in VSMC in culture and in the abdominal aorta of pigs fed an atherogenic diet that promoted neointimal thickening [53]. The inhibition of the isoprenylation of geranylgeranylated proteins underlies the effect of simvastatin, a response that was mimicked by inhibitors of RhoA and ROCK and by a RhoA dominant-negative [53]. Additionally, the agonist of the glucagon-like peptide-1 receptor, Exendin-4, inhibits both ERK1/2 signaling and NOR-1 expression reducing the rate of proliferating VSMC and limiting neointimal thickening induced by endothelial denudation [62].

The critical role of NOR-1 regulating proteins that are major players in cell cycle progression would explain why this NR is the target of several microRNAs relevant to vascular hyperplasia and remodeling. A miRNA microarray analysis in human aortic VSMC stimulated with PDGF, identified miR-638, a miRNA highly expressed in human VSMC, as one of the miRNAs most down-regulated by PDGF [60]. This study identified NOR-1 as a target of miR-638 and demonstrated that NOR-1 down-regulation underlies the miR-638-dependent inhibition of PDGF-induced VSMC proliferation and cyclin D1 expression. A similar role of miR-638 has been found in airway smooth muscle cells (ASMCs), whose proliferation and migration contribute to asthma [116]. Further, in VSMC from the pulmonary artery, miR-107 negatively modulates PDGF-induced cell proliferation and migration by targeting NOR-1 [117]. In fact, NOR-1 has emerged as a key transcription factor regulating both pulmonary artery VSMC proliferation induced by mitogens and vascular remodeling in response to hypoxia in chronic obstructive pulmonary disease (COPD) [80,118]. Several studies focused on NOR-1 as an essential gene targeted by miRNAs that controls pulmonary artery VSMC hyperplasia such as miR-638 [81], miR-508-3p [82] or miR-106b-5p [83], highlight this NR as a promising therapeutic target for the design of pharmacological strategies in pulmonary arterial hypertension and acute pulmonary embolism. Of note, resveratrol, that has been considered the magic bullet for pulmonary hypertension, limits pulmonary vascular remodeling regulating the NOR-1/cyclin D1 axis via miR-638 [81,119]. 

Besides essential genes/proteins of the cell cycle directly or indirectly regulated by NOR-1, in the last years, several structural genes involved in vascular remodeling have been identified as NOR-1 targets. Among them, it is worth highlighting vitronectin (VTN) [120], a multifunctional glycoprotein which participates in cell adhesion, migration, and proliferation as well as in thrombosis and fibrinolysis [121]. NOR-1 and VTN co-localize in human atherosclerotic lesions, and NOR-1-induced VTN contributes to VSMC migration [120]. Indeed, cell supernatants from VSMC over-expressing NOR-1 induced cell migration to a greater degree than those from control cells, an effect that was abolished by VTN blocking antibodies or VTN silencing [120]. Alpha-2-macroglobulin (A2M) a broad-spectrum proteinase inhibitor, which is mainly expressed in the liver, but also in the vascular wall, also deserves special mention. Indeed, NOR-1 can impact ECM remodeling by modulating MMP expression and activity by several mechanisms, including the regulation of A2M transcription [122]. Last but not least, NOR-1 modulates VSMC redox homeostasis through the control of enzymes involved in ROS production (NADPH oxidases NOX1 and NOX4) and scavenging (superoxide dismutase 1 (SOD1), SOD2, and SOD3). The over-expression of NOR-1 in VSMC increased ROS generation in parallel with a concomitant increase of the NADPH oxidase, NOX1 [63]. Both NOR-1 and NOX-1 co-localize in human atheroma and gene silencing, luciferase reporter, site-directed mutagenesis, and EMSA studies confirmed the regulation of NOX-1 transcription by NOR-1 [63]. Similarly, NOR-1 up-regulates SOD1 and SOD3, while it down-regulates NOX4 and SOD2, the latter by antagonizing NFκB signaling [63]. Therefore, NOR-1 orchestrates the complex and intricate network of genes involved in redox homeostasis in the vasculature. Additionally, recent insights in VSMC refer to NOR-1 in the induction of the long noncoding RNA Lnc-Ang164 (GIVER, Growth factor- and cytokine-induced vascular cell-expressed lncRNA) by Ang II [61]. NOR-1 and GIVER are regulated in a coordinated manner by Ang II, growth factors and pro-inflammatory cytokines, and their expression is increased in the arterial wall of hypertensive patients, supporting their implication in the pathogenesis of hypertension.

The up-regulation of NOR-1 by LPS, oxLDL, and cytokines, including IL-1β and TNFα, reduces the response of VSMC to these pro-inflammatory stimuli [18]. Certainly, lentiviral over-expression of NOR-1 in human VSMC reduces the basal expression of cytokines and chemokines (IL-1β, IL-6, IL-8, MCP-1 y CCL20), as well as their up-regulation by LPS, TNFα, or LDLox [18]. Accordingly, the acute inflammatory response elicited by LPS in the aortic wall was lower in NOR-1 transgenic mice (TgNOR-1^VSMC^) than in WT animals. This anti-inflammatory effect of NOR-1 resulted from the attenuated activation of mitogen-activated signaling pathways (ERK1/2, p38 MAPK and c-jun N-terminal kinase), that translated into decreased phosphorylation and degradation of IĸBα, and consequently, less activation of the NFκB pathway and its downstream effectors [18].

Finally, as detailed below in AAA, in VSMC NOR-1 is also induced by hypoxia [34]. In the vasculature from rats exposed to hyperbaric conditions that simulate diving, HIF1α was stabilized in medial VSMC, and a transcriptomic analysis found increased expression of NOR-1, as well as of genes previously identified as NR4A targets (plasminogen activator inhibitor 1 (PAI-1) and serpin 1) [123,124]. These data suggest that NOR-1 may be a relevant factor for the acute adaptation of the vasculature to changes in ambient pressure and breathing oxygen content. Further, it cannot be ruled out that the hypoxic microenvironment generated in atherosclerotic plaques and AAA [34,125] could account for the increased expression of NOR-1 documented in these diseased vessels. 

#### 3.1.3. NOR-1 in Monocytes/Macrophages 

Atherosclerosis entails a dysfunctional interplay between inflammation and lipid metabolism, in which monocytes/macrophages are major players [2]. Early studies from Dr. Tontonoz’s lab in monocytic/macrophage cell lines reported that several inflammatory stimuli (LPS, cytokines [TNFα, IL-1β and INFγ] and oxidized lipids [oxLDL, 25-hydroxycholesterol and 7β-hydroxycholesterol) strongly induced the expression of NR4A receptors through an NFκB-dependent mechanism [27]. Lentiviral over-expression of NR4A receptors in RAW264.7 and J774 macrophages led to increased expression of genes involved in the control of cell cycle, apoptosis and inflammation, including the inducible IκB kinase (IKKi/IKKϵ) an essential component of the NFκB pathway [69]. These results seemed to suggest a pro-inflammatory role of NR4A receptors. Subsequent studies, however, support that these receptors exert anti-inflammatory functions. All three NR4A receptors are expressed in infiltrating macrophages from human atherosclerotic lesions in both early and advanced stages, and are transiently up-regulated in THP-1 macrophages stimulated with LPS or TNFα [68]. Gain- and loss-of-function approaches in THP-1 revealed that these receptors attenuate macrophage activation and pro-inflammatory activity, reducing the production of cytokines and chemokines (IL1β, IL8, macrophage inflammatory protein-1α, [MIP-1α], MIP-1β and MCP-1), as well as the expression of the scavenger receptor type A (SR-A) and CD36, thereby limiting the uptake of oxLDL and the formation of foam-cells [68]. According to that, Qing and collaborators [46] showed that the specific deficiency of NOR-1 in hematopoietic stem cells accelerates atherosclerosis. Irradiated ApoE^−/−^ mice reconstituted with bone marrow-derived cells from NOR-1^−/−^ mice and fed a high fat diet showed increased plaque size and macrophage infiltration [46]. The authors further evidenced that NOR-1 deletion strongly increased the expression of the Runt-related transcription factor 1 (RUNX1), a transcription factor that suppresses NOR-1 and is a key gene in hematopoiesis [126]. This effect increased the proliferative activity of macrophages and dendritic progenitor cells in bone marrow, and induced Ly6C+ monocytosis. Further, in the vascular wall, NOR-1 knockdown increased foam cell formation within the atheroma. Therefore, these data indicate that NOR-1 limits atherosclerosis through a negative modulation of myelopoiesis in the stem cell compartment and by preventing the pro-atherogenic activity of macrophages in the vascular wall.

The anti-inflammatory role of NR4A receptors in general, and of NOR-1 in particular, is further supported by the findings that associate the expression of NOR-1 with M2 macrophage polarization, a process involved in the resolution of inflammation (Figure 2). In fact, IL-4-induced polarization of primary monocytes into alternative M2 macrophages induces NOR-1 expression, while NOR-1 knockdown reduces the expression of M2-specific markers, such as IL-10 and the interleukin-1 receptor antagonist (IL-1Ra) [127]. In agreement, in human atherosclerotic plaques, immunohistochemical studies evidenced that NOR-1 is strongly expressed in alternative macrophages (CD68+ MR+). A similar function seems to be played by Nur77, as Nur77 deficiency up-regulates M1-markers and shifts macrophages toward a pro-inflammatory phenotype [74]. A recent study reported an indirect mechanism through which ET-1 could regulate macrophage activation [128]. Conditioned medium from endothelial cells over-expressing ET-1 stimulated with oxLDL contains miR-33-loaded exosomes that once transferred to macrophages increased the expression of genes related to classical M1 macrophages while down-regulated NOR-1 as well as M2 macrophage markers. Altogether, this favors macrophage activation and the induction of pro-inflammatory genes. This mechanism of intercellular communication involving the miR-33/NR4A axis would contribute to the exacerbated atherosclerosis found in ApoE^−/−^ mice with endothelium-specific ET-1 overexpression [128]. Studies addressing the function of NR4A receptors in monocytes/macrophages in other pathological scenarios seem to confirm their role as regulators of alternative macrophage polarization and their anti-inflammatory effect [129,130,131]. 

The fine-tune regulation of NR4A receptors in monocytic lineage cells and their relevant role in metabolism [107], have led to propose NOR-1, together with PPARδ, another NR which modulates macrophage function and inflammation [132], as biomarkers of metabolic syndrome [133]. Indeed, Random Forest Analysis determined that the expression of both NOR-1 and PPARδ were specifically decreased in CD14+ cells (mainly monocytes) from patients with metabolic syndrome, identifying these patients with high specificity and sensitivity [133].

This anti-inflammatory and anti-atherogenic function, however, is somehow questioned by studies that found no differences in atherosclerosis and a comparable induction of genes associated with M1 and M2 phenotypes in control animals and in LDLR^−/−^ mice transplanted with bone marrow from mice deficient in NOR-1 or Nur77 [75]. Further, it has been suggested that, in plaque macrophages, NOR-1 would act as a mediator of the receptor-interacting protein kinase 3 (RIP3), a signaling molecule in the programmed necrosis pathway that regulates cytokine production, induces inflammation and exacerbates atherosclerosis [134,135]. 

#### 3.1.4. NOR-1 Controls T and B Lymphocyte Activity

NR4A receptors play a key role controlling peripheral T cell development and function (see for review [136]). Since the late 1990s, it is well-established the functional redundancy between NOR-1 and Nur77 during the process of clonal deletion that eliminates T-cell antigen receptor (TCR)-expressing immature thymocytes by apoptosis [137], a critical mechanism for the generation of a peripheral T-lymphocyte population with low potential autoreactivity. NR4A extranuclear functions seem to be involved this process. Certainly, both Nur77 and NOR-1 are phosphorylated by PKC and translocate to the mitochondria, where they interact with Bcl2 inducing the exposition of the pro-apoptotic BH3 domain [138,139]. NR4A receptors are also indispensable for the development of regulatory T cells (Treg), a T cell lineage critical for the control of self-tolerance. NR4A receptors, mainly Nur77 and NOR-1, directly activate the transcriptional activity of the transcription factor Foxp3, which controls the development, maintenance, and function of Treg cells [140]. The T cell-specific deficiency of NR4A receptors in mouse leads to a complete depletion of Treg cells and promotes the premature death of mice due to severe systemic autoimmunity. Therefore, these NRs essentially determine the fate of thymic CD4+ T cells and immune homeostasis. In fact, NR4A, which are highly expressed in Treg cells compared to other T cell subsets, regulate the expression of genes which specify Treg cell lineage and control its function [141]. 

In the last years, the major role of immunity in atherosclerosis and the impact of the Treg/Th17 balance on plaque initiation, progression, and rupture have become clear [142,143]. In this context, our group recently identified CD69 as a new receptor for oxLDL in T cells, indispensable for the induction of NR4A receptors and Treg differentiation [26]. Mice lacking CD69 on lymphoid cells fed an atherogenic diet show increased blood Th17/Treg ratio and exacerbated atherosclerosis. In fact, oxLDL binding to CD69 induces the expression of anti-inflammatory NR4A receptors, in particular NOR-1, and inhibits Th17 cell development (Figure 2). Further, peripheral blood leukocytes from individuals with subclinical atherosclerosis participating in the PESA (Progression of Early Subclinical Atherosclerosis) study displayed reduced CD69 and NR4A expression, supporting their usefulness as early atherosclerotic markers [26].

Similarly, B cells have also been involved in atherosclerosis. Different B cell subsets critically modulate inflammation and atherosclerosis through the production of antibodies and cytokines, exhibiting both atheroprotective and anti-atherogenic properties [144]. In particular, while B1 and marginal zone B (MZB) cells are considered as atheroprotective, follicular B cells promote atherosclerosis. The activation of B cells requires antigen stimulation (signal 1) which induces B cell proliferation and primes these cells to recruit, engage and respond to T cell help (signal 2). To trigger a productive immune response, and upon encounter with an antigen, B cells need T cell co-stimulation which helps to enforce self-tolerance. In this way, B cells die by apoptosis if signal 2 is not launched in a specific temporal window. It has recently been reported that NOR-1 and Nur77 are quickly induced by B cell antigen receptor (BCR) stimulation and act in a partially redundant manner to limit B cell responses to antigen in the absence of T cell co-stimulation [145]. This effect relies, at least in a part, on a NR4A-dependent repression of basic leucine zipper transcription factor ATF-like (BATF) and consequently of MYC, paired with reduced expression of chemokines such CCL3 and CCL4, as well as CD86 and ICAM-1 [145]. This mechanism constitutes a new negative feedback loop yielding B cells highly dependent on co-stimulation and avoiding that strongly activated B cells monopolize T cell help when this is limiting. Recently, in LDLR^−/−^/Nur77^−/−^ mice fed an atherogenic diet, it has been evidenced that complete B- or specific MZB-cell deletion of Nur77 increases atherosclerosis [77]. However, no studies have addressed whether the activity of NOR-1 in B cells impacts on atherosclerosis. 

#### 3.1.5. NOR-1 in Mast Cells, Neutrophils, and Dendritic Cells

Proteins and lipid mediators released by activated mast cells are new players recently recognized in vascular remodeling in atherosclerosis and AAA [146,147]. Mast cell activation specifically up-regulates NOR-1, while the expression of the other two NR4A members does not seem significantly affected [148]. In fact, NOR-1 was the most induced gene in mast cells infected with live streptococci [149], and also the main NR4A receptor up-regulated in these cells by IgE receptor cross-linking or after LPS stimulation [148]. Proteases such as chymase and tryptase, growth factors, histamine, and chemokines released by activated mast cells promote ECM degradation, vascular cell apoptosis and inflammatory cell recruitment into the vessel wall [150]. This process seems to be regulated by NOR-1 through the control of mast cell degranulation and thereby of cytokines/chemokines secretion [151]. However, currently, the functional implications that the induction of NOR-1 in these cells may have for atherosclerosis have not been investigated in detail, although these cells regulate innate and adaptive immune responses, vasodilatation, vascular homeostasis, and angiogenesis [152]. 

Likewise, neutrophils [147,153] and dendritic cells [147,154] have gained attention due to their involvement in vascular remodeling in atherosclerosis and AAA. Neutrophils are key elements in the innate immune response and constitute the first line of defense of the immune system. Defects in their number and survival characterize hematopoietic disorders and chronic inflammatory diseases. Further, through the release of neutrophil extracellular traps (NETs), neutrophils participate in the pathogenesis of thrombosis, atherosclerosis, and AAA [155,156]. Although neutrophils are short-lived cells, they can expand their lifespan. Protein kinase A (PKA) signaling controls neutrophil survival and microarray studies aiming to characterize the transcriptional response triggered by PKA activation in these cells identified both NOR-1 and Nurr1 as the most up-regulated genes associated with a delay in apoptosis [157]. Both receptors are highly expressed at sites of inflammation in a human model of intradermal inflammation coincident with neutrophil recruitment, while their knockdown limited apoptosis and prolonged neutrophil lifespan [157]. Regarding dendritic cells, they participate in all the stages of atherosclerosis and AAA due to the myriad functions that they play in immunity and tolerance induction, ranging from lipid uptake, efferocytosis, and antigen presentation to secretion of pro- and anti-inflammatory cytokines [147,154]. Initially, NOR-1 was associated with dendritic cell apoptosis [158]. However, subsequent studies supported that this receptor is involved in toll-like receptor (TLR)-mediated activation and gene expression induction [159], while the axis NOR-1/FOXO1/CCR7 seems to be critical for the migration of these cells [160]. More recently, NOR-1 has emerged as an essential transcription factor that guides monocyte differentiation toward monocyte-derived dendritic cells in response to microbes [161]. However, and despite the growing number of studies involving NOR-1 in the homeostasis of mast cells and dendritic cells, no specific studies have analyzed whether this NR modulates vascular remodeling through its function in these cells.

### 3.2. NOR-1 in AAA

Our group described for the first time the increased expression of NOR-1 in human aneurysmal samples from patients undergoing surgical repair [34]. Owing to the anti-inflammatory properties of this transcription factor in macrophages [46,68,127] and VSMC [18], it was hypothesized that NOR-1 could regulate the expression of genes with vasoprotective functions and that its up-regulation in the aneurysmal wall could be a compensatory mechanism to slow down the progressive arterial wall damage underlying this disease. With this premise, and considering that the specific deficiency of NOR-1 in hematopoietic stem cells accelerates atherosclerosis [46], Dr. Bruemmer’s group investigated whether the function of NOR-1 in the hematopoietic compartment impacts on AAA development [71]. LDLR^−/−^ irradiated mice were reconstituted with hematopoietic stem cells isolated from NOR-1 knockout animals and AAA was induced by Ang II infusion combined with a saturated fat-enriched diet. Although NOR-1 deletion modified the expression profile of inflammatory genes in macrophages, neither the in vivo echocardiographic follow-up, nor in vitro morphometric analyses showed any evidence that the specific deletion of NOR-1 in hematopoietic stem cells impacts on Ang II-induced AAA [71]. 

**Figure 4:**
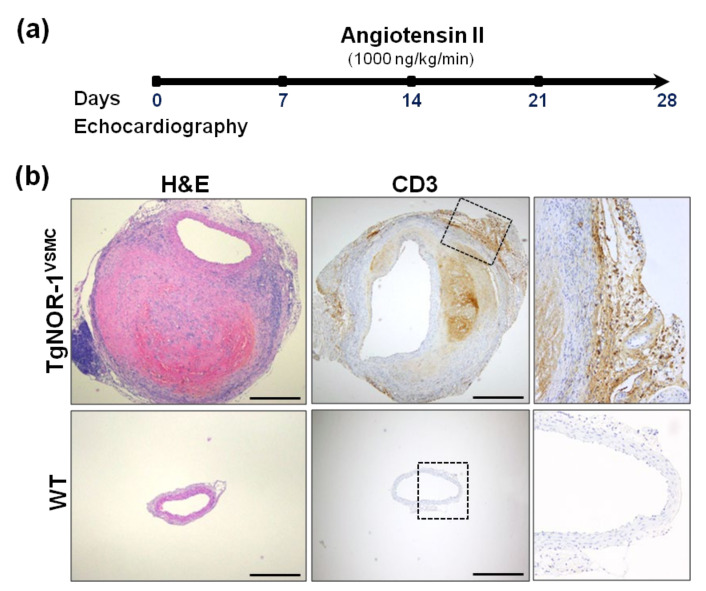
NOR-1 transgenesis predisposes to angiotensin (Ang II)-induced abdominal aortic aneurysm (AAA). (**a**) Experimental design to assess the role of NOR-1 on AAA. TgNOR-1^VSMC^ and wild-type (WT) mice were subjected to subcutaneous Ang II infusion. Aortic diameter was weekly assessed by echocardiography. After four weeks, aortas were excised and processed for further analysis. (**b**) The upper segment of the aneurysm was formalin-fixed and paraffin-embedded for histological and immunohistochemical analysis. Aortic sections from Ang II-infused mice were stained with hematoxylin-and-eosin (H&E) to visualize vascular remodeling (left panel) or subjected to immunohistochemistry to evidence the infiltration of CD3+ cells (lymphocytes; central and right panels). The indicated areas are magnified on the right. Bars: 500 µm. This figure is based on the studies that led to previously published data [78].

Since the human pathology courses with the increase of NOR-1 in the aneurysmal aorta [34], we were interested in assessing the impact of NOR-1 up-regulation in the pathogenesis of AAA. To reproduce this condition, we used two animal models that over-express human NOR-1 in the vascular wall (TgNOR-1 and TgNOR-1^VSMC^) [73,90]. Using this approach, we showed that high vascular NOR-1 expression strengthens the susceptibility to Ang II-induced AAA (Figure 4) [78]. Certainly, NOR-1 transgenesis amplifies the vascular response to Ang II, inducing a stronger vascular inflammatory response and exacerbating vascular wall disruption, oxidative stress, and MMP expression. The combined action of these responses overcame the natural resistance of the C57BL/6 strain to Ang II-induced AAA. Echocardiographic data showed that NOR-1 transgenesis favors an early vascular dilation, and the development of complex aneurysms after four weeks. The incidence and severity of AAA was similar in transgenic mice over-expressing NOR-1 in all cells of the vascular wall and in the hematopoietic compartment than in those animals in which NOR-1 specifically targets VSMC, thus supporting that the up-regulation of NOR-1 in medial VSMC is a necessary and sufficient condition to aggravate the vascular response to Ang II. Interestingly, doxycycline, a drug that prevents experimental aneurysms [162], reduces inflammation in human AAA [163] and has been tested in clinical trials [164], abrogated the formation of AAA in TgNOR-1^VSMC^ [78]. All together, these results highlight the relevant role of NOR-1 on AAA pathophysiology and support the usefulness of NOR-1 transgenic mice to strengthen our knowledge about disease mechanisms and as novel preclinical models for the screening of new pharmacological tools.

Particularly noteworthy was the identification of a large number of genes whose expression was modulated in the vascular wall of Ang II-infused TgNOR-1^VSMC^ mice [78]. Gene set enrichment analysis (GSEA) identified those signaling pathways and biological processes responsible for the higher AAA susceptibility induced by NOR-1. Among them, there were not only some pathways involved in inflammation/immune response, ECM remodeling, or VSMC differentiation, but also a group of genes classified in the category of “synaptic signaling”, whose expression was increased in aneurysms from TgNOR-1^VSMC^ mice. This category encompasses genes encoding for enzymes involved in the biosynthesis of catecholamines such as tyrosine hydroxylase (TH) and dopamine β-hydroxylase (DBH) [78]. The enhanced expression of TH and DBH was further detected in aneurysmal lesions from the Ang II-infused ApoE^−/−^ mouse model and, more importantly, in human aneurysmal samples, in which TH expression is more than 100 times higher than that of healthy aortas and correlates with NOR-1 mRNA levels [79]. TH expression localized mainly in peripheral sympathetic nerves innervating the vascular wall (GAP43+ cells), infiltrated lymphocytes (CD3+ cells), and scattered medial VSMC (SM α-actin+ cells). Notably, the TH competitive inhibitor metyrosine or α-methyl-p-tyrosine (AMPT) was able to prevent aortic dilation limiting Ang II-induced AAA in both TgNOR-1^VSMC^ and ApoE^−/−^ mice [78]. This drug drastically reduced the incidence and severity of aneurysms and ameliorated vascular damage triggered by Ang II by decreasing the infiltration of inflammatory cells, attenuating the expression of inflammatory markers such as MCP-1, preserving elastin fiber integrity and preventing the increase of ECM-degrading MMPs and oxidative stress [78]. Interestingly, transient transfection studies evidenced that NOR-1 regulates TH transcriptional activity through a NBRE site located in its promoter. Thus, our findings evidence that the inhibition of a NOR-1-target gene prevented AAA development, emphasizing the role of both NOR-1 in VSMC and the TH biosynthetic route in the pathophysiology of this disease. 

### 3.3. NOR-1 in Cardiac Remodeling 

In the last years, the members of the NR4A subfamily have emerged as key players in the pathophysiology of the heart. NOR-1 is highly expressed in the healthy heart in both animal models and humans [22,98,165]. This transcription factor is also expressed in the skeletal muscle [22,165], where it regulates the expression of genes involved in specific aspects of lipid, carbohydrate and energy homeostasis [40,100], and in the adaptive response of skeletal muscle to exercise, being one of the most exercise-responsive genes and driving muscle reprogramming in response to physical training [41,42,166]. In the heart, Nur77 has been extensively studied in different models of cardiac ischemia/reperfusion (IR) injury, acute myocardial infarction (AMI), and cardiac hypertrophy (for a review see [167]). However, much less is known about the role of NOR-1 in cardiac function and remodeling. NOR-1 expression is rapidly up-regulated by β-adrenergic stimulation in mouse heart [165] and neonatal rat cardiomyocytes (NRCMs) [89], associated with a concomitant regulation of the expression profile of genes involved cardiac metabolism. Moreover, NOR-1 contributes to isoproterenol-induced cardiomyocyte hypertrophy [89]. Studies in NRCMs evidenced that hypertrophy was attenuated by siRNA-mediated NOR-1 deletion, while NOR-1 over-expression potentiates NRCM hypertrophy [89]. This NOR-1-induced pro-hypertrophic response in NRCM seems to be mediated by the binding and activation of PARP-1 (Poly(ADP-Ribose) Polymerase 1), a DNA damage sensor that plays a pivotal role in the pathogenesis of several CVDs [168]. In fact, the inhibition of PARP-1 ameliorated the NRCMs hypertrophic response induced by NOR-1 [89]. Likewise, recent studies in an animal model generated by our group that over-express human NOR-1 in the heart (TgNOR-1) provided evidence that NOR-1 regulates key genes involved in cardiac function and myocardial remodeling in hypertensive cardiac hypertrophy [90]. Both cardiomyocytes and cardiofibroblast from NOR-1 transgenic mice exhibit important functional and gene expression alterations. Increased cell surface area, and higher expression of myosin heavy chain 7 (Myh7) and Myh7/Myh6 expression ratio were detected in TgNOR-1 cardiomyocytes. In agreement with the higher expression of the main contractile protein of the sarcomere, encoded by Myh7, these cells exhibited enhanced cell shortening in response to electric field stimulation. In turn, NOR-1 transgenesis promotes the phenotypic switch of fibroblasts to myofibroblasts, inducing higher collagen synthesis and migratory activity. Interestingly, TgNOR-1 mice showed an age-associated cardiac remodeling and a higher susceptibility to Ang II-induced cardiac hypertrophy and fibrosis. This response is characterized by the up-regulation of hypertrophic and fibrotic markers such as Myh7 and lysyl oxidase-like 2 (LOXL2), both of them identified as direct NOR-1 target genes [90]. Therefore, collectively, these data support a pivotal contribution of NOR-1 to the complex transcriptional program underlying hypertensive cardiac hypertrophy.

The available information regarding the contribution of NOR-1 to other cardiac processes such as IR injury and AMI is scarce. In humans undergoing coronary artery by-pass grafting, which promotes IR injury, cardiac NOR-1 expression is up-regulated [169]. Inflammatory/immune cells also play an important role in remodeling following acute myocardial injury [170,171]. In this regard, a recent study reported that lentiviral over-expression of NOR-1 by direct intra-myocardial injection reduced inflammation and limited infarct size and cardiac dysfunction after AMI [88]. This response is mediated by the NOR-1-dependent interference of NF-κB signaling, which reduces both IκBα phosphorylation and p65 nuclear translocation in a STAT3-dependent manner. Further, NOR-1 protects cardiomyocytes against the cellular stress induced by doxorubicin, an anti-tumoral drug which promotes cardiotoxicity [85]. It should be highlighted that NOR-1 has been identified as part of the whole blood molecular signature that discriminates patients with AMI [172], supporting its role as a biomarker in this disease. Likewise, it has been described that NOR-1 is located in the center of a functional gene cluster, essential for leukocyte activation and regulation of apoptosis, that influences the pathogenesis of peripheral CD146+ blood cells during the development of AMI [173]. Interestingly, atrial appendage transcriptional profile in patients with atrial fibrillation identified NOR-1 as one of the genes whose expression was most altered [91]. However, no study has investigated whether NR4A receptors play a role in atrial fibrillation, the most common arrhythmia, or in other arrhythmias. Finally, early microarray expression studies reporting NOR-1 up-regulation in the rat heart in response to exercise [174] suggested a role for this NR in the physiological adaptation of the heart to physical activity. However, and despite the well-studied contribution of NOR-1 to the exercise adaptation of skeletal muscle, no studies have addressed this issue on the cardiac adaptive response. Overall, although several studies point to NOR-1 as a potentially relevant NR regulating cardiac function and remodeling in a wide variety of pathological scenarios, the specific role of NOR-1 in the heart and in cardiac diseases is poorly understood.

## 4. Conclusions and Future Perspectives

The nuclear receptor NOR-1 seems to play a relevant role in regulating the function of vascular cells, cardiomyocytes, and inflammatory cells and the immune response in cardiovascular remodeling underlying atherosclerosis, AAA, pulmonary artery hypertension, and cardiac hypertrophy. The contribution of this transcription factor to VSMC activation/proliferation and neointimal thickening/vascular remodeling shown in different experimental models of vascular injury or pulmonary arterial hypertension should be highlighted. NOR-1 also mediates anti-inflammatory actions on cells from the monocyte–macrophage lineage and controls immune homeostasis through the regulation of Treg differentiation and function. However, it has also been suggested that it could mediate inflammation and exacerbate atherosclerosis. The impact of these somewhat paradoxical effects on the natural history of atherosclerosis in humans remains to be elucidated. It should be kept in mind that, as a transcription factor, NOR-1 is able to modulate many genes in different cells types and tissues, and depending on the specific pathophysiological context and the interplay with other regulatory pathways/transcription factors, the net impact of NOR-1 on global gene expression and cell function may significantly vary. In the last years, the application of “omics” technologies and the development of specific animal models have boosted the knowledge about the contribution of this NR4A receptor to the pathophysiology of cardiovascular remodeling. However, it is still unknown, whether NOR-1 function on B cells, neutrophils, mast cells, or dendritic cells impact on CVDs, while its relevance in cardiomyocyte homeostasis and cardiac dysfunction beyond cardiac hypertrophy should be unveiled. On the other hand, although several studies point to the expression of NOR-1 in circulating cells as a potential biomarker, these results should be validated in larger studies. In summary, what we currently know about the biology of NOR-1 and its potential clinical implications is much less than what we do not know about it. Taking into account the potential clinical implications, further studies are warranted to elucidate the specific responses and functions orchestrated by NOR-1 in the different cell types involved in CVDs, in order to establish their impact on the onset and progress or the eventual regression of pathological cardiovascular remodeling.

## Figures and Tables

**Table 1:** Involvement of NOR-1 and other NR4A receptors in pathophysiological processes and diseases in different organs and systems.

Organ/System	Pathophysiological Process/Pathology	References
Vascular wall	VSMC migration/proliferation	[19,22,24,25,53,54,55,56,57,58,59,60,61,62]
	VSMC redox homeostasis	[61,63]
	EC activation/survival/proliferation	[16,28,30,31,32,33,34,57,64,65]
	EC neovascularization	[66,67]
	Macrophage lipid uptake	[68]
	Inflammation	[16,18,27,57,61,68,69,70,71]
	Neointimal hyperplasia	[54,56,62,72,73]
	Atherosclerosis	[26,57,74,75,76,77]
	AAA	[78,79]
	PAH	[80,81,82,83]
Heart	Apoptosis/survival	[84,85]
	Calcium homeostasis	[86]
	IR injury	[84]
	Post-MI remodeling	[87,88]
	Ventricular hypertrophy	[86,89,90]
	Atrial fibrillation	[91]
Liver	Liver regeneration	[43]
	Hepatoprotection/fibrosis	[92]
	Glucose homeostasis/Gluconeogenesis	[39,93]
	IR injury	[94]
	Esteatosis	[95,96]
	Cancer	[93]
Brain	Dopamine synthesis	[97]
	Apoptosis	[98]
	Memory	[44]
	Neuroinflammation/IR injury	[17]
	Parkinson	[52]
Skeletal muscle	Insulin signaling/glucose metabolism	[49,95,99]
	Energy metabolism	[40,41,100]
	Muscle mass and hypertrophy	[42,100]
	Lipolysis	[101]
Pancreas	β-cell proliferation	[102]
	Insulin secretion	[103,104]
	Diabetes	[103]
	Cancer	[50]
WAT	Adipogenesis	[47]
	Insulin signaling	[49]
	Obesity	[48,95]
BAT	Thermogenesis	[105]
HPA axis	Steroidogenesis	[106]
	Regulation of ovulation	[29]

AAA: abdominal aortic aneurysm; BAT: brown adipose tissue; EC: endothelial cells; HPA axis: hypothalamic-pituitary-adrenal axis; IR: ischemia/reperfusion; MI: myocardial infarction; PAH: pulmonary arterial hypertension; VSMC: vascular smooth muscle cells; WAT: white adipose tissue.
